# Disease Prevention Not Decolonization: A Model for Fecal Microbiota Transplantation in Patients Colonized With Multidrug-resistant Organisms

**DOI:** 10.1093/cid/ciaa948

**Published:** 2020-07-18

**Authors:** Rohma Ghani, Benjamin H Mullish, Julie A K McDonald, Anan Ghazy, Horace R T Williams, Eimear T Brannigan, Siddharth Mookerjee, Giovanni Satta, Mark Gilchrist, Neill Duncan, Richard Corbett, Andrew J Innes, Jiří Pavlů, Mark R Thursz, Frances Davies, Julian R Marchesi

**Affiliations:** 1 Division of Digestive Diseases, Department of Metabolism, Digestion, and Reproduction, Imperial College London, London, United Kingdom; 2 Department of Infection, Imperial College Healthcare NHS Trust, London, United Kingdom; 3 Department of Gastroenterology and Hepatology, St Mary’s Hospital, Imperial College Healthcare NHS Trust, London, United Kingdom; 4 MRC Centre for Molecular Bacteriology and Infection, Imperial College London, London, United Kingdom; 5 Department of Renal Medicine, Hammersmith Hospital, Imperial College Healthcare NHS Trust, London, United Kingdom; 6 Centre for Haematology, Imperial College London, and Hammersmith Hospital, Imperial College London NHS Trust, London, United Kingdom; 7 School of Biosciences, Cardiff University, Cardiff, United Kingdom

**Keywords:** antimicrobial resistance, gut microbiome, fecal microbiota transplant, multidrug-resistant bacteria, allogeneic hematopoietic cell transplantation

## Abstract

Fecal microbiota transplantation (FMT) yields variable intestinal decolonization results for multidrug-resistant organisms (MDROs). This study showed significant reductions in antibiotic duration, bacteremia, and length of stay in 20 patients colonized/infected with MDRO receiving FMT (compared with pre-FMT history, and a matched group not receiving FMT), despite modest decolonization rates.

**(See the Editorial Commentary by Khanna on pages 1448–9.)**

Fecal microbiota transplantation (FMT) is an established treatment for recurrent *Clostridioides difficile* infection (rCDI), a pathogen proposed to colonize the same ecological niche within the intestinal microbiome as multidrug-resistant organisms (MDROs) [1, 2]. The use of FMT for the treatment of rCDI is recommended in international guidelines [3, 4]; furthermore, FMT has also been proposed to reduce intestinal colonization by MDROs, by restoring a patient’s disturbed microbiome with the diverse microbial community characterizing the gut microbiome of healthy stool donors [5].

Some patients with rCDI treated with FMT had a lower number and diversity of MDRO genes reported within the gut microbiome post-FMT [2]. To date, more than 193 patients have been in studies using FMT as treatment for MDROs [6], but study design has been heterogeneous. Decolonization has been the main endpoint, with eradication rates of 37.5–87.5% [6], overlapping with reported spontaneous decolonization rates of 48% [7]. Despite this, few studies [8–10] have explored the role of FMT in the prevention of infection and clinical impact on patients colonized with MDROs. Here we present an observational pre/post study of 20 patients with MDRO colonization or infection who received FMT to prevent disease occurrence/recurrence.

## METHODS

### Setting and Patient Selection

This study was performed between 2015 and 2019 in a London group of 5 hospital sites with approximately 1500 inpatient beds. The study was approved by a UK Research Ethics Committee (REC reference: 19/LO/0112). Multidrug-resistant organism was defined as vancomycin-resistant enterococci, carbapenem-producing *Enterobacteriaceae*, or extended-spectrum β-lactamase (ESBL)–producing *Enterobacteriaceae*.

Patients were selected according to 2 groups, as follows:

Group 1: Patients with intestinal colonization (rectal screening) with an MDRO and considered at risk of invasive MDRO disease. This included hematology patients with planned immunosuppression (ie, allogeneic hematopoietic cell transplantation [HCT]). Fecal microbiota transplantation would aim to take place at least 2 weeks before further immunosuppression.
Group 2: Patients with recurrent MDRO-mediated invasive disease and considered at risk of further disease. This included patients with recurrent urinary tract infections (UTIs)—in particular, renal transplantation patients where recurrent infection was adversely impacting graft function [11].

In both groups, FMT was scheduled when patients were not receiving antibiotic therapy and considered infection free. The aim of FMT in both groups was to prevent invasive MDRO infections.

Patients were observed for at least 6 months post-FMT and monitored for MDRO carriage, invasive infection (bloodstream infection [BSI] or UTI), number and days of intravenous and oral antibiotic courses, antibiotic susceptibility of invasive/colonizing isolates, and inpatient bed days.

### Donor Selection and Fecal Microbiota Transplantation Administration

Fecal microbiota transplantation was administered via nasogastric tube using prefrozen donor stool (see full details in Supplementary Methods).

### Outcome Metrics and Statistics

Days of antibiotic therapy, infection episodes, and length of stay in the 6 months pre- and post-FMT were recorded from clinical notes/electronic prescription charts. Multidrug-resistant organism decolonization was assessed by serial rectal swab or stool sample analysis for at least 6 months post-FMT via opportunistic screening at clinic appointments. Statistical analysis was performed by Wilcoxon ranked pairs, for nonparametric data, using GraphPad Prism 8.

### Comparator Group

A comparator arm analysis was also performed. Comparator patients had clinical profiles similar to both FMT groups, were treated over the same time period, and had previous infection/colonization with MDROs but were not considered for FMT (lead clinician or patient choice). Analysis was performed for the first and second 6 months from the first identified MDRO (also see Supplementary Methods).

## RESULTS

**Figure 1.**
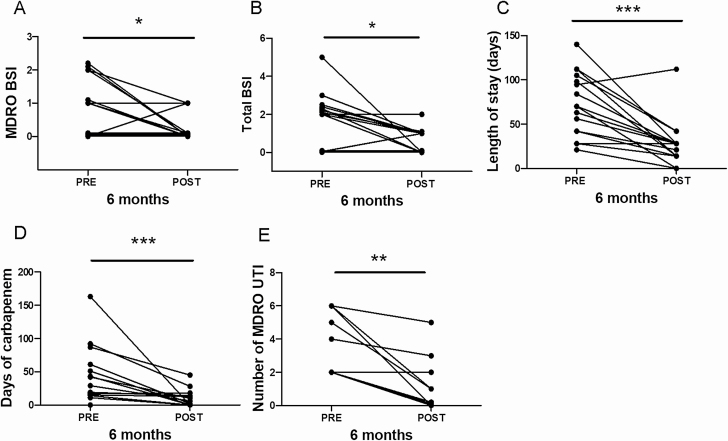
Clinical outcomes. *A*, Number of MDRO BSIs 6 months pre- and post-FMT (*P = .047; n = 20). *B*, Number of all BSIs 6 months pre- and post-FMT (*P = .03; n = 20). *C*, Length of inpatient stay (days) 6 months pre- and post-FMT (pre-FMT = 70 ± 35 days [median ± SD], post-FMT = 28 ± 26 days; ***P = .0002; n = 16; incomplete data available for 4 patients). *D*, Number of days of carbapenem use 6 months pre- and post-FMT (pre-FMT = 36 ± 44 days [median ± SD], post-FMT = 4 ± 13 days; ***P = .0005; n = 14; incomplete data available for 6 patients). *E*, Number of MDRO UTIs 6 months pre- and post-FMT in group 2 (pre-FMT median = 4 ± 2 episodes, post-FMT median = 1 ± 2 episodes; **P = .008; n = 9). Abbreviations: BSI, bloodstream infection; FMT, fecal microbiota transplantation; MDRO, multidrug-resistant organism; UTI, urinary tract infection.

Twenty patients were recruited to the study, 11 to group 1 (hematology) and 9 to group 2 (recurrent UTI). Patient characteristics are displayed in Supplementary Table 1. Across both groups, there was a significant reduction in BSI after FMT for both MDRO BSIs (*P* = .047) (Figure 1A) and all BSIs (*P* = .03) (n = 20) (Figure 1B). This reduction was not seen in the comparator group (BSI across all patients, *P* = .24; n = 40; MDRO BSI for group 1 comparator arm only, *P* = .28; n = 20) (Supplementary Figure 1). There was also a significant reduction in inpatient length of stay post-FMT in both groups (pre-FMT median = 70 ± 35 days, post-FMT median = 28 ± 26 days; *P* = .0002; n = 16) (Figure 1C); this was also not seen in comparator patients (*P* = .16, n = 40). Patients had significantly reduced carbapenem use post-FMT (pre-FMT median = 36 ± 44 days, post-FMT median = 4 ± 13 days; *P* = .0005; n = 14) (Figure 1D), which was again not seen in comparator patients (*P* = .61; n = 32). Seven of 17 (41%) patients were no longer colonized with MDROs on rectal screening following FMT (follow-up range: 6 weeks–24 months).

All patients tolerated FMT well with no serious adverse events. Mild adverse effects included self-limiting constipation, bloating, and diarrhea.

### Group 1 Subanalysis

See Supplementary Results 2.1 for full demographics of hematology FMT recipients. Post-FMT, 8 patients underwent allogeneic HCT. All patients had shorter inpatient stays (*P =* .002) and fewer days on carbapenems compared with the preceding 6 months (*P* = .002); this reduction was not seen in group 1 comparator patients (*P* = .48; n = 20). One patient undergoing HCT post-FMT developed an MDRO BSI caused by a different organism from their previous colonizing organism. This BSI was treated with a shorter course of antibiotics relative to their pre-FMT infections (42 days pre-FMT, 10 days post-FMT). In the group 1 comparator arm, 8 patients died during the 12 months of first identification of MDRO colonization.

### Group 2 Subanalysis

See Supplementary Results 2.1 for full demographics of recurrent UTI FMT recipients. There was a significant reduction in frequency of MDRO UTIs post-FMT (pre-FMT median = 4 ± 2 episodes, post-FMT median = 1 ± 2 episodes; *P* = .008; n = 9) (Figure 1E), which was not seen in the group 2 comparator arm (*P* = .18; n = 20). Only 1 patient with recurrent CDI/UTI coinfection developed a further ESBL UTI 6 months post-FMT. Three renal transplant patients had a marked reduction in days of antibiotics and both inpatient and outpatient attendances post-FMT. Two patients required inpatient antibiotic therapy immediately post-FMT for ESBL-driven infection (for both patients, urine collected at the time of FMT was culture-positive for ESBL organisms); this may have impacted the efficacy of the FMT. One patient underwent a second FMT after a 6-month interval, resulting in no further MDRO UTI during the study period.

## DISCUSSION

The major novel finding of our study is the significant post-FMT reductions in inpatient bed days, bacteremias, and antibiotic use in both cohorts; this finding is despite modest rates of intestinal decolonization, consistent with previously published reports [6] (also see Supplementary Discussion). Particularly notable was the reduction in BSI in hematology patients, where no patients developed bacteremia with their pre-FMT colonizing bacteria, despite ongoing systemically active chemotherapy/immunosuppression, including allogeneic HCT. This observation starkly contrasts with the group 1 comparator arm, where there was no reduction in BSI over time, and a marked number of deaths, findings that require further analysis. In patients with recurrent UTI, there was difficulty establishing an infection-free window in which to perform FMT for some patients. Nevertheless, there was a significant reduction in antibiotic use and use of oral antibiotics rather than intravenous. In 1 case, a second FMT was performed, with improved effectiveness; this approach may be a model for such patients in the future.

However, this study has several limitations, and some clinically important questions remain unanswered. With regard to limitations, the patient cohort was small and nonrandomized. Infection was not fully prevented in the renal group, and factors such as structural abnormality post–renal transplantation may have been factors. However, delayed onset of efficacy of FMT seems to be apparent and requires further work to be fully understood. For the hematology cohort, chemotherapy was usually different pre- and post-FMT, and larger studies are essential to exclude confounders such as chemotherapeutic agent choice. Antibiotic choices varied widely between patients, and the broader impact of FMT on use of World Health Organization “Restrict” and “Watch” group antibiotics merits exploration in future studies. The mechanisms of FMT are incompletely understood; however, improvement in colonization resistance by microbiome restoration in “at risk” groups appears to be a more important factor then full intestinal eradication of MDROs when assessed in terms of patient outcome.

### Conclusions

These data support and extend upon prior studies demonstrating that FMT presents a novel antimicrobial stewardship intervention in patients colonized with intestinal MDROs. Particular considerations are required for the use of FMT in this group of patients that are not of such relevance when FMT is being used in those with rCDI (eg, care is needed to exclude pre-existing infections, particularly in those with recurrent UTI). Patients should be discussed in a multi-disciplinary team (MDT) setting with experienced clinicians, regarding modifying external factors to prevent reinfection, as well as timing of FMT (ensuring enough time between further immunosuppression and stopping long-term antibiotics). There should be a modified expectation of outcomes based on the premorbid state of the patient, and a move away from expecting full intestinal decolonization. This study demonstrates that, in carefully selected patients, FMT offers a hope for reduction in MDRO infections and the associated morbidity.

## Supplementary Data

Supplementary materials are available at *Clinical Infectious Diseases* online. Consisting of data provided by the authors to benefit the reader, the posted materials are not copyedited and are the sole responsibility of the authors, so questions or comments should be addressed to the corresponding author.

ciaa948_suppl_Supplmentary_Material

## Notes

***Financial support:*** The Department of Metabolism, Digestion, and Reproduction and the Centre for Haematology at Imperial College London receive funding from the National Institute of Health Research (NIHR) (United Kingdom), Imperial Biomedical Research Centre (United Kingdom), Medical Research Council (United Kingdom), and Wellcome Trust (United Kingdom). B. H. M. and A. J. I. are the recipients of NIHR Academic Clinical Lectureships, and B. H. M. was previously the recipient of a Medical Research Council (MRC) Clinical Research Training Fellowship (grant reference: MR/R000875/1). J. A. K. M. is the recipient of a Wellcome Trust Institutional Strategic Support Fund (ISSF) fellowship. F. D. receives funding from the MRC Clinical Academic Research Partnership Scheme.

***Potential conflicts of interest:*** B. H. M. reports personal fees from Finch Therapeutics Group, outside the submitted work. All other authors report no potential conflicts. All authors have submitted the ICMJE Form for Disclosure of Potential Conflicts of Interest. Conflicts that the editors consider relevant to the content of the manuscript have been disclosed.
