# Supplementary material for: Disease Prevention Not Decolonization: A Model for Fecal Microbiota Transplantation in Patients Colonized With Multidrug-resistant Organisms
Source: Clin Infect Dis. 2020 Jul 18;72(8):1444–7. doi: 10.1093/cid/ciaa948 (PMC8075030; doi:10.1093/cid/ciaa948)
Supplement: ciaa948_suppl_Supplmentary_Material [file ciaa948_suppl_supplmentary_material.docx]

**Supplementary Material for:**

**Brief report**

**Disease prevention not decolonization – a model for fecal microbiota transplantation for patients colonized with multidrug-resistant organisms**

1. **Supplementary Methods:**
   1. **FMT administration protocol:**

This was standardized for all FMT procedures, and followed the best practice principles described in the UK joint British Society of Gastroenterology/ Healthcare Infection Society FMT guidelines [1,2]. All procedures and follow-up were performed prior to the Covid-19 pandemic [3].

FMT planning involved a multidisciplinary specialist team. All FMT procedures endorsed by the local hospital Antibiotic Review Group. CMV IgG negative donors were used to prevent CMV reactivation/disease [4]. In accordance with current UK guidance, all donors were screened at every donation for CPE, VRE and ESBL, to prevent introduction of new MDROs [5].

All antibiotics being administered for clinical care were discontinued for at least 24 hours prior to the FMT procedure; no specific ‘pre-FMT’ antibiotic regimen was routinely administered. FMT recipients all received bowel purgatives on the day prior to the procedure, using a polyethylene glycol (PEG)-based preparation. Patients were administered oral proton pump inhibitor on the night prior to and the morning of the procedure, and also received metoclopramide approximately 30 minutes prior to FMT administration. Nasogastric tube insertion was performed either on the night before or morning of the procedure, with positioning confirmed using chest radiograph.

The FMT used in all procedures had been previously prepared in house anaerobically and stored at -80^o^C for less than six months, using 10% w/v glycerol as cryopreservative [6,7]. Each FMT unit had been prepared from at least 50 grams of crude stool. FMT was thawed on the morning of the procedure at 4^o^C; when thawed, FMT was diluted to 100ml, and drawn into syringes ready for administration.

- 1. **Comparator Arm Analysis:**

For Group 1/ hematology patients, comparator patients were selected from a list of patients who were colonised on rectal screening or who had an MDRO bacteremia between 2015-2019, and had not received FMT; age and sex matching to the FMT group was performed as much as possible. 20 patients were identified in this group. Medical notes were examined for: number of BSI (total and MDRO); days of carbapenem therapy (where available); and length of stay. The notes were examined for a 12-month period from date of first MDRO organism identification, and split into 0-6 and 6-12 months from date of MDRO, for analysis purposes.

For the Group 2/ recurrent UTI comparator arm, 20 patients were selected from a list of patients who had more than 4 MDRO episodes of UTI per year from 2015-2019; again, age and sex matching to the FMT group was performed as much as possible. These were patients who were under the care of renal or urology services and who had not undergone urological surgery or other interventions in the 12 months from the date of first MDRO UTI. The medical notes were examined for: the number of MDRO urinary tract infections; number of BSI (total and MDRO); days of carbapenem therapy (where available); and length of stay. Again, data were split into 0-6 and 6-12 months from date of MDRO, for analysis purposes.

Wilcoxon signed pairs rank test was used to assess statistical significance between the two time periods for each patient.

1. **Supplementary Results:**
   1. **Baseline characteristics of Group 1 and Group 2 FMT recipients:**

*Group 1:*

Of 11 patients with an underlying hematologic disorder, six had an MDRO BSI pre-FMT, and nine patients had prolonged admissions (range 6-20 weeks) complicated by septic episodes. Patients were colonized with CPE (8), VRE (3) or ESBL (2) as detected on rectal screening.

*Group 2:*

Nine patients underwent FMT for recurrent ESBL UTIs (seven patients with *Escherichia coli* and two *Klebsiella pneumoniae*). This included four patients with rCDI co-infection and five patients with renal transplantation. The renal transplant patients had each required inpatient stay of >2 months duration within the preceding year with infection-related graft dysfunction; three patients had received multiple prolonged courses of intravenous antibiotics administered via the outpatient parenteral antibiotic team.

**2.2 Comparator arm baseline characteristics and analysis**

20 patients were included for hematology and 20 for recurrent UTI analysis; the results are displayed in **Supplementary Figure 1**. 12 of the 20 comparator Group 1/ hematology patients had further chemotherapy, including allogeneic (4) or autologous (1) stem cell transplantation during the 12 months of analysis after their first isolated MDRO. Eight patients were diagnosed with MDRO colonisation or infection during their admission for stem cell transplantation. Seven of the 20 comparator Group 1/ hematology patients died within the 12 month study period from date of first MDRO, one of whom received an 11 month course of outpatient parenteral tigecycline prior to death.

Of the 20 comparator Group 2/ recurrent UTI patients, all were under active follow-up with either renal or urology services, and included 18 patients with previous renal transplantation.

There were no significant differences seen over time in any of the clinical domains analyzed in either of the comparator groups examined (**Supplementary Figure 1**).

1. **Supplementary Discussion:**

Our data regarding the impact of fecal microbiota transplant (FMT) upon recurrent urinary tract infection (UTI) extend upon those of prior studies. For instance, Tariq and colleagues described eight patients with recurrent *Clostridioides difficile* infection (CDI) and recurrent UTI who received FMT; they noted a reduction in frequency of UTI post-FMT, and improved antibiotic susceptibility of UTI-causing bacteria [8]. None of these patients were reported as having received renal transplantation or being immunosuppressed [8]. The outcome measures presented in our study are different, focussing on the clinical impact of MDRO infections, in terms of event number, length of stay and defined days of carbapenem administration. Due to the wide variety of available antibiotics available for administration, and difficulty measuring compliance with out-patient oral antibiotic administration, carbapenems only were measured, rather than total antibiotic consumption. As such, our finding that FMT appears to provide similar clinical benefit in this particular cohort, even without prior CDI, extends the potential therapeutic scope of this treatment modality.

There has also been prior exploration of the use of FMT for the attempted decolonization of antibiotic-resistant bacteria from the gut microbiome of patients with chronic hematological disease. The largest such study to date was provided by Bilinski and colleagues, who administered 25 FMTs (via the upper gastrointestinal route) to 20 such patients [9]. This study demonstrated intestinal decolonization of antibiotic-resistant bacteria occurring in 60% of FMTs by one month post-intervention (with highest rates on decolonization occurring in patients where there was no peri-procedural use of antibiotics). The particularly high rate of decolonization noted in this study may reflect any of a number of distinctive factors about this work, including the particular clinical nature of the patients included, or aspect related to the mechanics of administration (i.e. using a large amount of stool (100g) for each FMT administration, with most patients receiving FMT on two consecutive days.

It is interesting to note that there are comparable findings to ours here within the FMT/ rCDI literature. In particular, Ianiro and colleagues assessed for the occurrence of bloodstream infection (BSI) within 90 days for patients with rCDI treated with either antibiotics or FMT [10]. On analysis of a subset of their cohort matched for baseline characteristics, it was demonstrated that risk of BSI was 23% lower in the FMT-treated patients, and that FMT-treated patients also had a significantly reduced length of hospitalization and extended overall survival compared to antibiotic-treated patients [10]. The mechanisms underlying these findings remain unclear, but may include FMT-related maintenance or restitution of gut barrier function, and/or gut microbiota-induced priming effects upon the host immune system, that could influence response to future pathogen exposure [11]. However, further experimental study is required to explore the contribution of these possible mechanisms *in vivo*.

**Supplementary Table 1: Patients’ underlying condition and associated MDRO.**

| **Patient Demographics** | | **Underlying diagnosis** | **Colonising/invasive organism** |
| --- | --- | --- | --- |
| **Group 1** | 33M | Acute myeloid leukaemia | *E. coli* ESBL (meropenem resistant) |
|  | 68F | Acute myeloid leukaemia | *E. coli* NDM |
|  | 68M | Acute myeloid leukaemia | *E. coli* GES5 |
|  | 59M | Chronic myeloid leukaemia | *C. freundii* OXA-48 |
|  | 63M | Mycosis fungoides | *K. pneumoniae* OXA-48 |
|  | 54M | Diffuse large B cell lymphoma | *C. freundii* OXA-48  Vancomycin resistant *enterococci* |
|  | 17F | Sickle cell disease and gut GVHD | Vancomycin resistant *enterococci* |
|  | 70M | Acute myeloid leukaemia | Vancomycin resistant *enterococci* |
|  | 63M | Acute lymphoblastic leukaemia | *K. oxytoca* GES5 |
|  | 55M | Chronic myeloid leukaemia | *E. coli* IMP-1 |
|  | 59M | Acute myeloid leukaemia | *K. pneumoniae* OXA-48 |
| **Group 2** | 89F | Recurrent UTI/ CDI | *E. coli* ESBL  *C. difficile* |
|  | 90F | Recurrent UTI/CDI | *E. coli* ESBL  *C. difficile* |
|  | 80F | Recurrent UTI/CDI | *E. coli* ESBL  *C. difficile* |
|  | 90F | Recurrent UTI/CDI | *E. coli* ESBL  *C. difficile* |
|  | 59F | Recurrent UTI/Renal transplant | *K. pneumoniae* ESBL |
|  | 62M | Recurrent UTI/Renal transplant | *E. coli* ESBL |
|  | 60F | Recurrent UTI/Renal transplant | *E. coli* ESBL |
|  | 51F | Recurrent UTI/Renal transplant | *K. pneumoniae* ESBL |
|  | 78M | Recurrent UTI/Renal transplant | *E. coli* ESBL  *C. freundii* OXA-48 |

**Supplementary Figure 1: Comparator arm of hematology and recurrent UTI patients infected or colonized with MDRO who did not receive FMT.** There was no significance difference seen in the first 6 months since first MDRO isolate (0-6) compared to the second 6 months (6-12) for: **A)** the number of MDRO BSI in comparator Group 1/ hematology patients (*P* = .28, *n*=20); **B)** total number of BSI, comparator Group 1/ hematology and Group 2/ recurrent UTI groups (*P* = .24 *n*=40); **C)** length of stay, comparator Group 1/ hematology and Group 2/ recurrent UTI groups (*P =* .16, *n*=40); **D)** days of carbapenem therapy, comparator Group 1/ hematology and Group 2/ recurrent UTI groups (*P =* .16, *n*=32; full data unavailable for 8 patients); **E)** the number of positive MDRO urine samples in comparator Group 2/ recurrent UTI patients (*P* = .18, *n*=20). NS: non-significant.


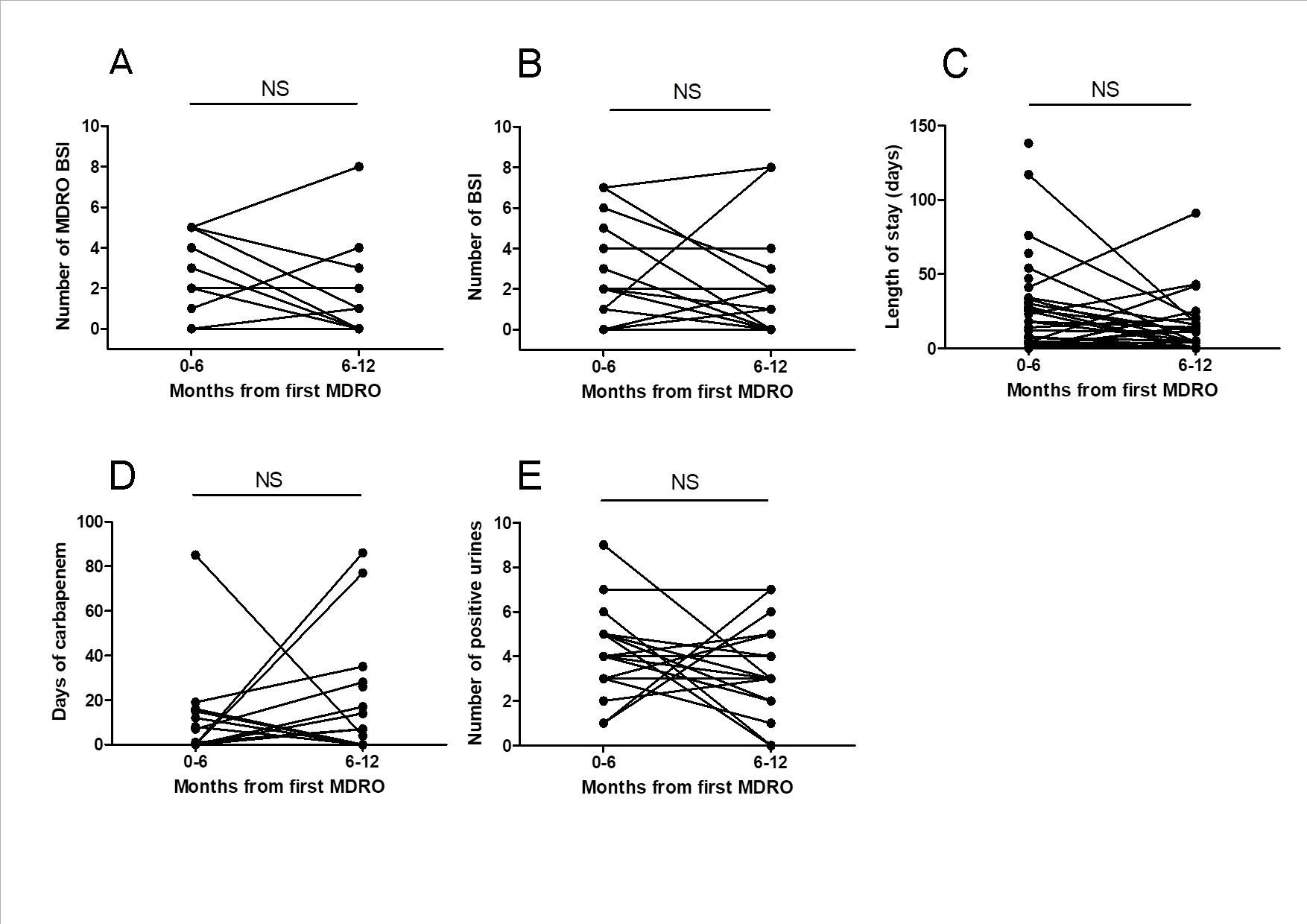


**References:**

1. Mullish BH, Quraishi MN, Segal JP, et al. The use of faecal microbiota transplant as treatment for recurrent or refractory Clostridium difficile infection and other potential indications: Joint British Society of Gastroenterology (BSG) and Healthcare Infection Society (HIS) guidelines. Gut **2018**; 67:1920–1941. Available at: http://gut.bmj.com/lookup/doi/10.1136/gutjnl-2018-316818. Accessed 5 September 2018.

2. Mullish BH, Quraishi MN, Segal JP, et al. The use of faecal microbiota transplant as treatment for recurrent or refractory Clostridium difficile infection and other potential indications: joint British Society of Gastroenterology (BSG) and Healthcare Infection Society (HIS) guidelines. J Hosp Infect **2018**; 100:S1–S31. Available at: https://linkinghub.elsevier.com/retrieve/pii/S019567011830402X.

3. Ianiro G, Mullish BH, Kelly CR, et al. Screening of faecal microbiota transplant donors during the COVID-19 outbreak: suggestions for urgent updates from an international expert panel. Lancet Gastroenterol. Hepatol. 2020; Available at: www.thelancet.com/gastrohep. Accessed 11 April 2020.

4. Cheng Y-W, Phelps E, Ganapini V, et al. Fecal microbiota transplantation for the treatment of recurrent and severe *Clostridium difficile* infection in solid organ transplant recipients: A multicenter experience. Am J Transplant **2019**; 19:501–511. Available at: http://www.ncbi.nlm.nih.gov/pubmed/30085388. Accessed 17 February 2019.

5. DeFilipp Z, Bloom PP, Torres Soto M, et al. Drug-Resistant E. coli Bacteremia Transmitted by Fecal Microbiota Transplant. N Engl J Med **2019**; Available at: http://www.ncbi.nlm.nih.gov/pubmed/31665575. Accessed 16 November 2019.

6. Mullish BH, Marchesi JR, Thursz MR, Williams HRT. Microbiome manipulation with faecal microbiome transplantation as a therapeutic strategy in Clostridium difficile infection. QJM **2015**; 108:355–359. Available at: https://academic.oup.com/qjmed/article-lookup/doi/10.1093/qjmed/hcu182. Accessed 20 March 2017.

7. Cammarota G, Ianiro G, Kelly CR, et al. International consensus conference on stool banking for faecal microbiota transplantation in clinical practice. Gut **2019**; 68:gutjnl-2019-319548. Available at: http://gut.bmj.com/lookup/doi/10.1136/gutjnl-2019-319548. Accessed 29 September 2019.

8. Tariq R, Pardi DS, Tosh PK, Walker RC, Razonable RR, Khanna S. Fecal Microbiota Transplantation for Recurrent Clostridium difficile Infection Reduces Recurrent Urinary Tract Infection Frequency. Clin Infect Dis **2017**; 65:1745–1747. Available at: http://academic.oup.com/cid/article/65/10/1745/3978076. Accessed 19 January 2020.

9. Bilinski J, Grzesiowski P, Sorensen N, et al. Fecal Microbiota Transplantation in Patients With Blood Disorders Inhibits Gut Colonization With Antibiotic-Resistant Bacteria: Results of a Prospective, Single-Center Study. Clin Infect Dis **2017**; 65:364–370. Available at: http://academic.oup.com/cid/article/65/3/364/3088486/Fecal-Microbiota-Transplantation-in-Patients-With. Accessed 19 January 2020.

10. Ianiro G, Murri R, Sciumè GD, et al. Incidence of bloodstream infections, length of hospital stay, and survival in patients with recurrent clostridioides difficile infection treated with fecal microbiota transplantation or antibiotics a prospective cohort study. Ann Intern Med **2019**; 171:695–702. Available at: http://www.ncbi.nlm.nih.gov/pubmed/31683278. Accessed 11 December 2019.

11. Quraishi MN, Shaheen W, Oo YH, Iqbal TH. Immunological mechanisms underpinning faecal microbiota transplantation for the treatment of inflammatory bowel disease. Clin. Exp. Immunol. 2020; 199:24–38.
